# Protein sorting from endosomes to the TGN

**DOI:** 10.3389/fcell.2023.1140605

**Published:** 2023-02-21

**Authors:** Dominik P. Buser, Anne Spang

**Affiliations:** Biozentrum, University of Basel, Basel, Switzerland

**Keywords:** retrograde transport, TGN, endosome, AP-1, clathrin, retromer, SNX, Rab9

## Abstract

Retrograde transport from endosomes to the trans-Golgi network is essential for recycling of protein and lipid cargoes to counterbalance anterograde membrane traffic. Protein cargo subjected to retrograde traffic include lysosomal acid-hydrolase receptors, SNARE proteins, processing enzymes, nutrient transporters, a variety of other transmembrane proteins, and some extracellular non-host proteins such as viral, plant, and bacterial toxins. Efficient delivery of these protein cargo molecules depends on sorting machineries selectively recognizing and concentrating them for their directed retrograde transport from endosomal compartments. In this review, we outline the different retrograde transport pathways governed by various sorting machineries involved in endosome-to-TGN transport. In addition, we discuss how this transport route can be analyzed experimentally.

## Introduction

Retrograde transport of lipids or proteins from the plasma membrane and endosomes to the trans-Golgi network (TGN) is crucial for membrane homeostasis and to retrieve components of anterograde transport machineries. Proteins recycled back to the TGN encompass transport receptors for soluble lysosomal acid-hydrolases, processing enzymes, SNAREs (soluble N-ethylmaleimide-sensitive fusion factor attachment receptors), nutrient transporters, and a subset of other intracellular transmembrane proteins with diverse functions (Bonifacino and Rojas, 2006). In addition, extracellular bacterial and plant toxins as well as viral proteins harness the retrograde transport route of host cells often hijacking host cell’s intrinsic factors (Sandvig and van Deurs, 1999; Johannes and Goud, 2000; Spooner et al., 2006). In fact, the analysis of such toxins led to the discovery and description of retrograde transport pathways involved in endosome-to-Golgi transport (Olsnes and Pihl, 1972; Montanaro et al., 1973; Gonatas et al., 1975). The idea that host factors might potentially shuttle these toxins into cells stimulated the search for endogenous client proteins for retrograde transport. Almost half a century later, not only several host transmembrane proteins have been identified, but also the underlying sorting machineries regulating transport from endosomes to the TGN. This review summarizes the current findings of the molecular machineries driving transport from endosomal membranes to the TGN. First, we will give an outline of the proteins that are subjected to retrograde traffic from endosomes before discussing the sorting devices regulating their transport.

## Retrograde cargo proteins

Efficient transport from endosomes to the TGN is restricted to a subset of transmembrane proteins that cycle between these two compartments. Retrograde cargo proteins vary considerably in their function and structure, but they can be basically grouped into five different classes: cargo receptors, processing enzymes, SNAREs, nutrient transporters, and other transmembrane proteins. The last category comprises a diverse set of integral membrane proteins whose function is unknown or different to the other classes, such as the trans-Golgi network integral membrane proteins (e.g., TGN46). In addition to these categories, viral, bacterial or plant toxins can be considered retrograde cargo proteins, however, they constitute a group of exogeneous rather than endogeneous cargo (Table 1). Attempts to provide a global overview of cargo proteins undergoing retrograde transport to the TGN have been made (Shi et al., 2012; Shin et al., 2020); systematic or in-depth analyses on the subjet are still scarce.

**TABLE 1:** Selection of cargo proteins that undergo retrograde transport from endosomes to the TGN.

Cargo class	Cargo protein	Functional information and references
Cargo receptors	CDMPR	Transport of lysosomal acid-hydrolases (Ghosh et al., 2003a)
	CIMPR	Transport of lysosomal acid-hydrolases (Ghosh et al., 2003a)
	Sortilin	Transport of soluble and transmembrane cargo (Gustafsen et al., 2013)
	SorLA	Transport of soluble and transmembrane cargo (Gustafsen et al., 2013)
	WLS	Transport of Wnt ligands (Hayat et al., 2022)
Integral membrane proteases	Furin	Subtilisin-like endopeptidase (Molloy et al., 1999)
	Carboxypeptidase D	Metallocarboxypeptidase (Varlamov and Fricker, 1998)
	BACE1/2	Processing of APP (Zhang and Song, 2013)
SNAREs	Syntaxin 5	SNARE involved in MPR transport (Amessou et al., 2007)
	Syntaxin 16	SNARE involved in MPR transport (Amessou et al., 2007)
	vti1a	SNARE involved in MPR transport (Amessou et al., 2007)
Nutrient transporters	GLUT4	Glucose transporter (Shi and Kandror, 2005)
	ATP7A/B	Copper transporter (La Fontaine and Mercer, 2007)
	DMT1-II	Iron transporter (Tabuchi et al., 2010)
	ANK	Unknown function (Seifert et al., 2016)
Other transmembrane proteins	TGN38/46/48/51	Unknown function (Mallet and Maxfield, 1999)
	APP	Unknown function (Choy et al., 2012)
Protein toxins	Shiga toxin	Inhibiton of translation (Sandvig and van Deurs, 2002)
	Cholera toxin	Regulation of adenylyl cyclase (Matsudaira et al., 2015)
	Ricin	Inhibiton of translation (Sandvig and van Deurs, 2002)
	Abrin	Inhibiton of translation (Sandvig and van Deurs, 2002)

Cation-dependent/-independent mannose-6-phosphate receptor (CDMPR/CIMPR); sortilin-related receptor with LDLR class A repeats (SorLA); Wntless (WLS); ß-site APP cleavage enzyme 1/2 (BACE1/2); vesicle transport through interaction with t-SNARE homolog 1a/b (vti1a/b); glucose transporter 4 (GLUT4); Menkes protein or ATPase copper transporting alpha/beta (ATP7A/B); divalent metal ion transporter 1-II; progressive ankylosis protein (ANK); trans-Golgi network integral membrane protein (TGN protein); amyloid precursor protein (APP).

### Cargo receptors

**FIGURE 1:**
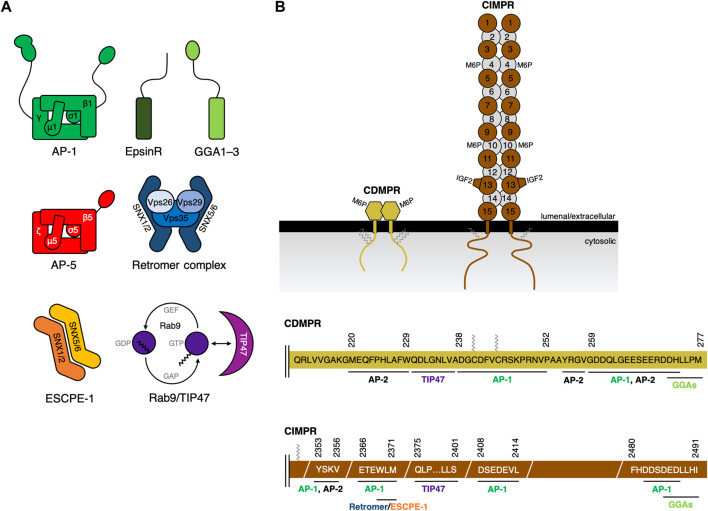
Sorting machinery proteins and cargo binding. **(A)** Schematic representation of sorting machinery proteins, including the clathrin adaptors (AP-1, epsinR, and GGAs), AP-5, the retromer complex, ESCPE-1, and Rab9/TIP47. Individual subunits are indicated by name. Note that ESCPE-1 particularly refers to the SNX1/SNX2-SNX5/SNX6 dimer. Other combinations of SNXs lead to additional ESCPE complexes. **(B)** Among the cargo for sorting machineries are the cation-dependent mannose-6-phosphate receptor (CDMPR) and the cation-independent mannose-6-phosphate receptor (CIMPR). Both CDMPR and CIMPR are present predominantly as stable homodimers in membranes. Both receptors have a different number of M6P-binding sites per polypeptide chain. CIMPR has further a binding site for IGF2. Both receptors can undergo various posttranslational modifications (e.g., palmitoylation) that can direct the receptor’s trafficking itinerary. A schematic representation of the cytosolic tails of the human MPRs, showing the predicted or identified amino-acid sorting signals and their associated transport proteins (sites for AP-2 are highlighted as reference as well). The residues after the transmembrane (TM) domain of CDMPR and CIMPR are indicated. Palmitoylation sites are indicated in gray. While CDMPR’s full cytosolic tail is depicted, only critical sites are highlighted for CIMPR. The drawn MPR tails are not proportional to each other in size. Adaptor protein complex 1/2 (AP-1/2); adaptor protein complex 5 (AP-5); Golgi-localized, γ-adaptin ear-containing, ARF-binding proteins 1-3 (GGA1–3); epsin-related protein (epsinR); tail-interacting protein 47 (TIP47); endosomal SNX-BAR sorting complex for promoting exit (ESCPE); vacuolar protein sorting (Vps) protein 26, 29, and 35 (Vps26, 29, and 35); sorting nexin (SNX); Bin/Amphiphysin/Rvs (BAR); spastic paraplegia proteins 11 and 15 (SPG11 and SPG15); Insulin-like growth factor 2 (IGF2). The MPR illustration is derived from another article (Ghosh et al., 2003a).

One of the most thoroughly studied cargo receptors cycling between endosomes and the TGN are the cation-dependent and -independent mannose-6-phosphate (M6P) receptors (CDMPR/MPR46 and CIMPR/MPR300), essential for efficient export of M6P-tagged lysosomal acid-hydrolases from the TGN (Griffiths et al., 1988; Kornfeld, 1992) (Table 1; Figure 1). Following cargo unloading in the mildly acidic endosomal environment, MPRs are recycled back to the TGN for reuse. Both MPRs exist in homodimers and display type I transmembrane topology, but they differ in size and abundance. While CDMPR is ∼46 kDa with an estimated copy number of ∼660,000 in HeLa cells, CIMPR is considerably larger with a molecular mass of ∼300 kDa but half as abundant (∼310,000 receptor molecules per cell) (Hirst et al., 2012b; Itzhak et al., 2016). The main difference in size is due to the more complex extracellular domain of CIMPR (Figure 1B). CIMPR, also known as IGF2R, does not only mediate binding of M6P-tagged acid-hydrolase for their lysosomal delivery, but plays also a role in internalizing insulin-like growth factor binding protein 2 (IGF2), a ligand critically controlling proper embryonic development (Ghosh et al., 2003a). Immunogold labeling studies have shown that the bulk of MPRs localizes to the TGN, endosomes, and the plasma membrane (PM) (Ghosh et al., 2003b; van Meel and Klumperman, 2014). Ten % of CIMPR and CDMPR are surface-localized at steady-state (Johnson et al., 1990; Ghosh et al., 2003b). The physiological importance of receptor cell surface localization, besides CIMPR’s role in IGF2 endocytosis, is mainly to recapture missorted M6P-tagged cargo. Cell surface-associated CIMPR has been recognized as efficient and potential therapeutic platform for targeted degradation of extracellular and transmembrane proteins using hexasaccharide–anti-target antibody conjugates by shuttling the target to lysosomes while CIMPR is retrieved to the TGN or plasma membrane for reuse (Banik et al., 2020).

Correct membrane targeting of MPRs is conferred by sorting signals present in their cytoplasmic tails. These sorting motifs, however, are different between CDMPR and CIMPR, and they might be therefore differently recognized by the diverse sorting machineries as discussed below (Ghosh et al., 2003a). Given their similar steady-state localization, it is assumed that both MPRs have redundant functions. The two MPRs, however, are not completely functionally redundant since mouse embryonic fibroblasts deficient of both receptors can only restore in part proper targeting of distinct lysosomal hydrolases when either CDMPR or CIMPR is separately overexpressed (Ludwig et al., 1993; Pohlmann et al., 1995; Kasper et al., 1996; Munier-Lehmann et al., 1996).

Yeast *Saccharomyces cerevisiae* has also an MPR-like receptor, Vps10, involved in directing cargo to the vacuole, demonstrating the important and evolutionary conserved function of the pathway.

Apart from the MPRs in mammalian cells, other recycling receptors such as sortilin and the sortilin-related receptor SorLA have proposed functions in sorting and escorting soluble and transmembrane cargo from the TGN (Nielsen et al., 2007; Fjorback et al., 2012; Gustafsen et al., 2013; Schmidt et al., 2017).

Another recycling cargo receptor undergoing retrograde transport from endosomes to the TGN is the integral membrane protein WLS (also known as Wntless, Evi, and GRP177) (Table 1). WLS transports Wnt proteins through the secretory pathway for their release at the cell surface (Harterink and Korswagen, 2012; Mittermeier and Virshup, 2022). Cargoless WLS is retrieved from the cell surface for additional rounds of cargo capture and release. At steady-state, WLS localizes to the endoplasmic reticulum (ER), Golgi complex, and the plasma membrane, suggesting that it might traffic back and forth between the ER and the plasma membrane. Indeed, WLS has been described to undergo not only retrograde transport to the TGN (Harterink et al., 2011), but in addition also retrieval to the ER (Yu et al., 2014). Efficient escorting of Wnt ligands by WLS for secretion thus does not occur at the level of the TGN, but already in the ER. WLS is therefore one of only a few endogenous receptors known to undergo retrograde plasma membrane-to-ER transport.

Whether the transferrin receptor (TfR), which imports iron into the cell *via* its ligand transferrin, undergoes endosome-to-TGN transport remains a matter of debate (Snider and Rogers, 1985; Shi et al., 2012). Most recent data suggest that TfR normally does not recycle through the TGN (Buser et al., 2018; Buser and Spiess, 2019).

### Integral membrane proteases

Integral membrane proteases include proprotein convertases that cycle between the TGN and the endo-lysosomal system. These enzymes typically have a type I membrane topology with an N-terminal lumenal protease domain that processes proprotein precursor domains of immature proteins, a transmembrane domain, and a cytoplasmic tail containing sorting determinants for targeted transport (Burd, 2011). Furin and carboxypeptidase D belong to this category of retrograde transport cargo (Varlamov and Fricker, 1998; Chia et al., 2011) (Table 1). Although these enzymes predominantly localize to the TGN, they also escape to endosomes. It is conceivable that cycling of the convertases contributes to their functionality. It is not known, however, whether these proteases are actively concentrated into TGN-derived carriers, as it is the case for MPRs at the TGN, or whether they simply leak out and need to be actively retrieved. Other members of this category of cargo proteins are the membrane proteases BACE1/2, enzymes involved in the processing of the amyloid precursor protein (APP) (Chow et al., 2010; O'Brien and Wong, 2011; Wahle et al., 2005).

### SNAREs

SNARE proteins constitute a large protein superfamily with more than 60 members in mammals (Hong, 2005; Sudhof and Rizo, 2011; Wang et al., 2017). SNAREs are type II transmembrane proteins of 20–30 kDa in size that are characterized by a C-terminal hydrophobic region that functions as membrane anchor. The function of the N-terminal portion of the SNAREs is to mediate membrane fusion. In the basic model of SNARE function, transport carriers that bud from the TGN carry specific vesicle-SNAREs (v-SNAREs) that interact with endosomal target-SNAREs (t-SNAREs) to mediate membrane fusion between the TGN donor membrane and the endosome acceptor membrane. After disassembly of the v-/t-SNARE complex, the v-SNARE must be recycled to the TGN. Thus, SNAREs are critical for the trafficking and membrane flow of many other proteins as they regulate membrane fusion. Since v-/t-SNARE complexes are not supposed to be promiscuous, SNAREs confer specificity to recycling pathways. Efficient endosome-to-TGN traffic of MPRs requires syntaxin 16 and vti1a (Medigeshi and Schu, 2003; Saint-Pol et al., 2004; Amessou et al., 2007) (Table 1).

### Nutrient transporters

The localization of nutrient transporters is mainly regulated by metabolic cues. This regulation optimizes the capacity of nutrient uptake, sustains intracellular nutrient homeostasis and also protects the cell from toxic amounts of nutrients (Burd, 2011). Retrograde transport cargo proteins belonging to this class are for instance GLUT4, Menkes proteins and DMT1-II (Table 1). GLUT4 transporter is translocated to the cell surface in insulin-responsive cells where it facilitates glucose uptake. Decreasing levels of glucose, and thus of insulin triggers, causes the nutrient transporter to undergo retrograde transport and storage in GLUT4 storage compartments (GSCs). GSCs are produced from the TGN (Bryant et al., 2002; Shi and Kandror, 2005; Jedrychowski et al., 2010). While GLUT4 trafficking for glucose uptake is governed by insulin levels, Menkes proteins (also known as ATP7A/B) are part of the mammalian copper transport pathway in which they continuously cycle between the Golgi complex and the plasma membrane (La Fontaine and Mercer, 2007; Polishchuk and Lutsenko, 2013). In cells sensing low extracellular copper concentrations, exit of the transporter from the TGN is slower than retrograde retrieval from the cell surface. Menkes copper transporters thus localize to the TGN in steady-state. When, however, the copper concentrations are increased, the rate of Menkes protein cell surface localization increases in parallel. Augmented cell surface expression of the transporter probably improves the efficiency of copper removal from cells (Petris and Mercer, 1999).

Divalent metal transporter 1-II (DMT1-II) is another member of the family of nutrient transporters. It operates in the transport of divalent metal ions, including iron, from the lumen of compartments into the cytosol (Garrick et al., 2003; Tabuchi et al., 2010). Even though TfR and DMT1-II functionally cooperate in iron uptake, they have distinct retrograde sorting itineraries. While TfR is recycled to the cell surface from early endosomes, DMT1-II undergoes first retrograde transport to the TGN and is then delivered back to the plasma membrane (Tabuchi et al., 2010). Different sorting itineraries of TfR and DMT1-II might provide a mechanism to avoid iron toxicity. Thus, retrograde transport is very important to regulate nutrient homeostasis.

Recently, a novel potential cargo cycling through the TGN has been described: the progressive ankylosis protein ANK, which is a predicted PP_i_ transporter (Seifert et al., 2016).

### Other transmembrane proteins

This category includes all kind of integral membrane proteins that cannot be classified as any cargo group described above. Among these are the trans-Golgi network integral membrane protein TGN46 and its isoforms (TGN38, TGN48, and TGN51) and APP (Table 1).

At steady-state, TGN46 and its isoforms exclusively localize to the TGN, suggesting that these cargoes are TGN-resident proteins. A number of studies showed, however, that TGN46 and its isoforms are also present at the cell surface from where they can be retrieved *via* endosomes back to the TGN (Rajasekaran et al., 1994; Ponnambalam and Banting, 1996; Ponnambalam et al., 1996; Banting and Ponnambalam, 1997; Banting et al., 1998; Mallet and Maxfield, 1999; Buser et al., 2018; Buser and Spiess, 2019). The biological relevance of this cycling between the TGN and the plasma membrane remains unclear because the function of TGN46 and its isoforms has not been revealed yet. Recently, also the TGN-derived carriers involved in anterograde transport of TGN46 and isoforms have been described (Wakana et al., 2012; Wakana et al., 2013; Wakana et al., 2015; Lujan et al., 2022), and the sorting machinery mediating their retrograde traffic from endosomes (Saint-Pol et al., 2004; Lieu et al., 2007; Lieu and Gleeson, 2010). Also, the ‘Golgi-resident proteins’ such as galactosyltransferase or sialyltransferase can escape the Golgi compartment. Recent data revealed these enzymes to be sorted out of the TGN. There is, however, no evidence of their retrieval to the Golgi yet (Podinovskaia et al., 2021; Sun et al., 2021). Thus, it is also conceivable that this escape is part of the regular turnover of these proteins and that they are degraded in lysosomes.

APP is also a type I membrane protein and a well-characterized cargo molecule, mainly due to its association with Alzheimer’s disease. APP localizes to endosomes and to the TGN in steady-state where it faces different processing enzymes (Choy et al., 2012). The biological role of APP in the cell is still unknown and remains to be determined. Since some of APP’s cleavage is reported to occur in the TGN, retrograde transport from endosomes to this compartment is required for proper enzymatic processing by γ-secretase (Burgos et al., 2010; Choy et al., 2012; Yoshida and Hasegawa, 2022). SorLA has been described to escort APP from endosomes to the TGN (Fjorback et al., 2012).

### Exogeneous non-host proteins

Apart from endogenous cargo, a subgroup of toxins and viral proteins also enter cells by retrograde transport from the cell surface (Bonifacino and Rojas, 2006; Johannes and Popoff, 2008). Protein toxins can be secreted for example, by bacteria (e.g., Shiga and cholera toxin) and plants (e.g., ricin and abrin). Most of these toxins have a modular organization. A ligand moiety that confers binding to cell surface glycoproteins or glycosphingolipids and an enzymatically active domain that inhibits host cell reactions in the cytosol. After binding to the cell surface, the toxins are internalized and reach endosomes either by clathrin-mediated or clathrin-independent endocytosis (CME or CIE). Some toxins, such as Shiga toxin, traverse the TGN and the Golgi complex to reach the ER, where the ligand and enzymatic domain then separate from each other. The enzymatic moiety gains access to the cytosol by retrotranslocation where it exerts its toxicity (Johannes and Goud, 1998; Johannes and Goud, 2000; Sandvig and van Deurs, 2002; Sandvig and van Deurs, 2005).

However, not all toxins undergo passage through the TGN. It was recently shown that the *pseudomonas* exotoxin (PE) A takes a novel endosomal route *via* nuclear-associated endosomes (NAEs) to reach the host nucleoplasm (Chaumet et al., 2015). Some endogenous cargo proteins, including some cell surface receptors, seem to follow this route as well (Shah et al., 2019).

## Sorting machineries involved in retrograde transport from endosomes

**FIGURE 2:**
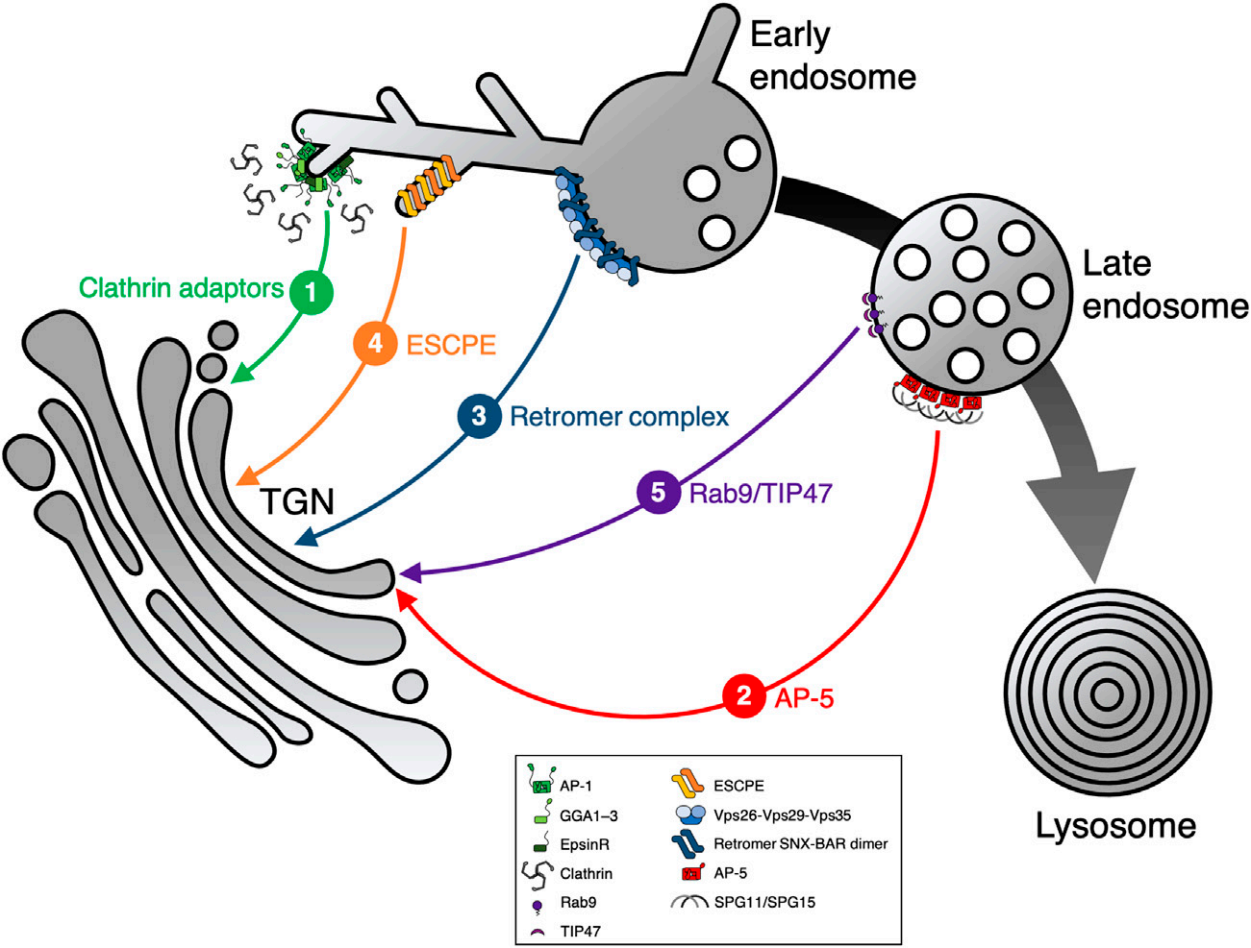
Sorting machineries involved in endosome-to-TGN transport. Following cargo internalization *via* endocytosis, several pathways exist that sort cargo protein from endosomes to the TGN. Cargo protein sorting can either occur from early or late endosomes, mediated by different sorting machineries: 1) clathrin adaptors, 2) AP-5, 3) retromer complex, 4) ESCPE (including ESCPE-1), and 5) Rab9/TIP47. All these pathways operate in parallel, though the extent of cooperation remains unknown. Clathrin adaptors include AP-1, GGA1–3, and epsinR. The large black/gray arrow highlights endosome maturation, thin colored lines represent individual transport pathways mediated by machinery as indicated (1–5).

After internalization by CME or CIE, cargo reaches early endosomes where it is either subjected to surface recycling, sorted further along the endo-lysosomal pathway for degradation, or transported to the TGN (Huotari and Helenius, 2011). Although the termination ‘early endosome’ is used differently in certain context among various articles, early endosome in our review includes both sorting (vacuolar) and tubular/recycling domains. Endosome-to-TGN transport is not only occurring from tubular early endosomes, but has been reported to occur during the entire endosome maturation process and also from late endosomes (Podinovskaia et al., 2021). As for other intracellular pathways, protein transport between these endocytic compartments and the TGN requires the formation, fission, and fusion of membrane-enclosed transport carriers. Molecular machinery components needed for the formation of selective transport carriers must be recruited from the cytosol to specific domains of the endosomal membrane to confer retrograde transport of cargo (Figure 1A; Figure 2).

### Clathrin adaptor-dependent pathway

Clathrin adaptors are a diverse set of monomeric and multimeric components of clathrin-coated carriers. These adaptors form an inner membrane-proximal coat that binds cargo, followed by the recruitment of an outer membrane-distal layer of clathrin. The Clathrin adaptors involved in intracellular endosome-to-TGN traffic are the adaptor protein complex 1 (AP-1), epsin-related adaptor protein (epsinR), and the Golgi-localized, γ-adaptin ear-containing, ARF-binding proteins 1-3 (GGA1–3) (Hirst and Robinson, 1998; Boman et al., 2000; Robinson and Bonifacino, 2001; Hirst et al., 2003). A picture has emerged over the years in which the different adaptors are no longer viewed as representatives of different pathways, but that they rather operate in concert and are found in stoichiometric ratios on a single clathrin-coated vesicle (CCV) (Hirst et al., 2012a; Hirst et al., 2015a). This idea was supported by super-resolution microscopy and comparative proteomics (Hirst et al., 2012a; Hirst et al., 2015a; Huang et al., 2019). Nevertheless, it is still possible that AP-1, epsinR, and GGAs are also able to form individual transport containers.

### AP-1

Adaptor protein complex 1 (AP-1) is a member of the heterotetrameric cargo adaptor protein (AP) complex family, a family that also comprises AP-2–5 (Figure 1A). Each of the five AP complexes localizes to a distinct intracellular compartment and has specific, but in part also overlapping cargo recognition function. In their role as cargo adaptors, they are recruited to their target membranes to mediate formation of specific transport carriers containing selected cargo proteins. The evolutionary tree of APs shows AP-5 diverging first, followed by AP-3 and AP-4, while AP-1 and AP-2 are most closely related (Hirst et al., 2014; Dacks and Robinson, 2017). AP-1 mainly localizes to the TGN and to tubular early endosomes, indicating the involvement in distinct sorting pathways (Meyer et al., 2000; Robinson et al., 2010; Bonifacino, 2014) (Figure 2).

Each of the AP complexes is composed of two distinct large subunits (∼100–130 kDa) termed β(1-5)-adaptin and γ-, α-, δ-, ε-, or ζ-adaptin, a medium μ(1-5)-subunit (∼50 kDa), and a small σ(1-5)- subunit (∼20 kDa). In the case of AP-1, the complex is made up of a β1-, γ-, μ1-, and σ1-adaptin subunit. The presence of individual AP-1 adaptin isoforms is cell type-specific. AP-1 has three σ1-adaptin isoforms (σ1A, σ1B, σ1C) (Boehm and Bonifacino, 2001). In polarized cells, for instance, there are two different μ1 subunits, μ1A and μ1B, with overlapping, but also separate functions (Folsch et al., 2003; Zizioli et al., 2010; Gravotta et al., 2012; Bonifacino, 2014). Recently, a second γ-subunit has been characterized, thereby giving rise to a γ1-and γ2-isoform-containing AP-1 complex (Zizioli et al., 2017). While the AP-1/γ1 complex is expressed in all eukaryotes, AP-1/γ2 expression only occurs in vertebrates and plants. They both have different functions in development and traffic and cannot functionally compensate for each other (Zizioli et al., 2017). To avoid any confusion when talking about the γ-Subunit of AP-1, we always refer to the ubiquitous γ1 (Lewin et al., 1998; Takatsu et al., 1998; Zizioli et al., 1999; Zizioli et al., 2017). The two large subunits (γ and β1) can be structurally subdivided into a C-terminal ear-like appendage domain connected to the N-terminal core domain *via* a flexible and unstructured linker sequence (Kirchhausen, 1999; Owen, 2004; Robinson, 2004; Canagarajah et al., 2013).

Membrane recruitment of AP-1 is dependent on activated ARF GTPases and is stabilized by binding to tyrosine- or dileucine-based sorting signals on cargo proteins *via* the μ1 subunit or the γ/σ1-hemicomplex (Stamnes and Rothman, 1993; Ohno et al., 1995; Seaman et al., 1996; Owen and Evans, 1998; Doray et al., 2007). While AP-1 and AP-2 functionally cooperate with clathrin and are thus *bona fide* components of clathrin-coated carriers (CCVs), this is less clear for AP-3. AP-4 and AP-5 use other coats (Robinson, 2004; Hirst et al., 2012b). Next to AP-2, which is exclusively required for endocytosis at the plasma membrane, AP-1 is the second most abundant cargo adaptor with a copy number of about 370,000 per cell (Hirst et al., 2012b).

As already indicated by its localization, AP-1 shuttles between the TGN and endosomes and thereby promotes recycling of cargo receptors such as of MPRs (Puertollano et al., 2001a; Ghosh et al., 2003a; Ghosh et al., 2003b; Puertollano et al., 2003). The steady-state distribution of both CDMPR and CIMPR in μ1A subunit-deficient fibroblasts derived from knockout mouse embryos was shifted to early endosomes at the expense of the TGN (Meyer et al., 2000). If AP-1 exclusively mediated anterograde transport from the TGN to endosomes, one would expect that in AP-1 knockouts both MPRs would accumulate in the TGN. MPRs, however, do exit the TGN in the absence of AP-1, reach the cell surface and are re-endocytosed from there, but accumulate to some extent in EEA1-positive compartments. Biochemical evidence for defective MPR retrieval from endosomes to the TGN was provided using a resialylation assay (Meyer et al., 2000). It was further demonstrated that membranes isolated from AP-1-deficient cells have a reduced transport competence in an *in vitro* retrograde endosome-to-TGN transport assay (Medigeshi and Schu, 2003). Both of these observations support a potential role of AP-1 in retrograde endosome-to-TGN traffic. Another readout for AP-1 dysfunction is the general observation that the lysosomal hydrolase precursor cathepsin D is preferentially missorted into the medium rather than being delivered to lysosomes (Meyer et al., 2000; Hirst et al., 2003; Hirst et al., 2005; Mardones et al., 2007). If M6P-mediated lysosomal hydrolase delivery was strictly dependent on MPRs for TGN exit, then also these cargo proteins should accumulate in the TGN, however, they do not. Lysosomal hydrolases exit the TGN independently of AP-1 and MPR localization, most likely through an AP-3-dependent pathway from the TGN to lysosomes. Apart from sorting of MPRs in the retrograde direction, other endocytosed cargo (e.g., cholera toxin) has been described to require AP-1 to reach the TGN (Matsudaira et al., 2015).

It is now generally accepted that AP-1 mediates bidirectional traffic between the TGN and endosomes, a role which is conserved from yeast to mammals (Meyer et al., 2000; Valdivia et al., 2002; Robinson et al., 2010; Anton-Plagaro et al., 2021). However, most of what we know about the involvement of AP-1 in bidirectional traffic at the TGN-to-endosome interface in mammalian cells is based on knockdown and knockout studies. A major disadvantage of gradual or long-term protein depletion strategies is that compensatory or indirect effects may occur, either due to cellular adaptation or altered steady-state distribution of factors involved in membrane fusion, for instance. In particular, in the case of a cargo adaptor like AP-1, which permanently shuttles proteins between two intracellular compartments, the question about directionality is more challenging to tackle, since the observed phenotype might not be a direct consequence of AP-1 dysfunction. Using a novel strategy for rapid protein inactivation termed knocksideways, the function of AP-1 in bidirectional traffic at the TGN-to-endosome interface was readdressed (Robinson et al., 2010). Using such a technique that eliminates possible compensatory effects, it could not only be confirmed that AP-1 functions in endosome-to-TGN transport of CIMPR, but also highlighted that gradual depletion (knockdown) and rapid depletion (knocksideways) can result in different phenotypes. While the protein levels of CIMPR present in clathrin-coated vesicles (CCVs) were substantially reduced in the knocksideways condition, almost no reduction of CIMPR levels was observed in the knockdown condition (Robinson et al., 2010). This study revealed that compensatory effects, such as the use of alternative APs, help the cell to cope with the loss of individual components. Moreover, the study raised the awareness of compensation in endosomal trafficking pathways and that acute perturbance might be a better strategy to reveal mechanistic insights compared to long-term depletion.

Using rapid depletion by knocksideways, the importance of AP-1 in endosome-to-TGN transport of EGFP-CIMPR and -CDMPR could be more quantitatively assayed by functionalized nanobodies directed against the fluorophore (Buser et al., 2018). When functionalized with a motif for tyrosine sulfation, these nanobodies allowed to mark surface MPRs and monitor their arrival in the TGN. Rapidly depleting AP-1 caused reduced sulfation of nanobodies, suggesting delayed transport to the TGN. Using the knocksideways approach to study the adaptor complex was necessary since depletion of AP-1 by knockdown or knockout caused, conversely, nanobody hypersulfation, an indirect phenotype of AP-1 complex removal (Buser et al., 2022). The observation of transport impairment of CIMPR and CDMPR to the TGN using derivatized nanobodies was another evidence for AP-1 operating in the retrograde direction (Buser et al., 2018).

Both MPRs depend on AP-1 to be shuttled back to TGN. The requirements to bind AP-1 for retrograde traffic seem to be similar for both receptors. Their cytoplasmic tails comprise a dileucine motif (Ghosh et al., 2003a). Disrupting the signal present in CDMPR by mutating the leucines to alanines causes a shift of receptor distribution from the perinuclear area to peripheral Rab5-positive compartments (Tikkanen et al., 2000). A similar receptor dispersal to peripheral membranes was also observed for CIMPR (Lobel et al., 1989; Tortorella et al., 2007), a phenotype as observed previously (Meyer et al., 2000). Palmitoylation/depalmitoylation of the tail of CDMPR and CIMPR has been documented to be another sorting determinant regulating their localization to endosomes and the TGN (Nair et al., 2003; Stockli and Rohrer, 2004; McCormick et al., 2008). An overview to which sequence of the cytosolic tail of CDMPR and CIMPR AP-1 and other machinery is binding is depicted in Figure 1B.

The notion of AP-1 to be involved in retrograde transport, or bidirectional transport in general, was elegantly corroborated in a quantitative proteomics study (Hirst et al., 2012a). The study demonstrated that AP-1 in cooperation with the adaptor GGA2 facilitates cargo sorting of lysosomal proteins with their receptors (e.g., CDMPR, CIMPR, etc.) for anterograde transport, while AP-1 alone operates in the selective retrieval of the empty cargo receptors in the retrograde direction (Hirst et al., 2012a). The idea of the orchestrated action of AP-1 and GGA adaptors at the TGN has been already suggested previously (Doray et al., 2002). While a link to the kinesin motor machinery driving AP-1-positive vesicles to move in the anterograde directon is established (*via* gadkin) (Schmidt et al., 2009; Laulagnier et al., 2011; Hirst et al., 2015a), a link to dynein family proteins regulating retrograde vesicle traffic is still missing.

Recently, an interaction of AP-1 with the transmembrane protein stimulator of interferon genes (STING or TMEM173) has been reported (Liu et al., 2022). Since this study focused on AP-1’s anterograde function in STING signaling, a possible role in receptor retriveal cannot be formally excluded. Not surprisingly, the absence of AP-1 function and associated factors has been described to be linked to multiple human disorders (Sanger et al., 2019; Duncan, 2022).

AP-1 has several reported accessory proteins. The first AP-1 binding partner identified was γ-synergin, a protein isolated in a yeast two-hybrid library screen for proteins that interacted with γ-adaptin (Page et al., 1999). A number of other AP-1-binding proteins have been identified by GST pulldowns using the γ-ear domain as bait or by database screening for sequences containing the γ-ear motif. Two of these proteins found in this manner were p200 and aftiphilin (Lui et al., 2003; Mills et al., 2003; Mattera et al., 2004). All of these components were shown to localize to AP-1-structures to different extents. Further, it was demonstrated that aftiphilin, p200, and γ-synergin form a complex, as evidenced by gel filtration, coimmunoprecipation, and RNAi experiments. While γ-synergin knockdown shows only weak phenotypes, those of aftiphilin and p200 mimicked the ones of AP-1, although less severe. Interestingly, knocking down AP-1 and the aftiphilin complex had opposing effects on transferrin recycling, however. Aftiphilin depletion led to the accumulation of transferrin in early endosomes (Hirst et al., 2005). The role and function of the aftiphilin/p200/γ-synergin complex in retrograde transport remains to be deciphered.

While we were mainly discussing the role of AP-1 in mediating bidirectional traffic at the TGN-to-endosome interface in polarized cells, it has to be mentioned that AP-1 is also involved in cargo recycling from endosomes to the plasma membrane in polarized cells. As mentioned before, polarized cells, such as epithelial cells or neurons, have two μ1-adaptins, μ1A and μ1B, giving rise to AP-1A and AP-1B. Accumlating evidence by different studies demonstrated that μ1A and μ1B play partly complementary roles in basolateral sorting, but that AP-1A might be mainly involved in biosynthetic sorting at the TGN and AP-1B in recycling to the basolateral surface from recycling endosomes (Folsch et al., 1999; Folsch et al., 2003; Gravotta et al., 2012; Bonifacino, 2014). Less is known about the involvement of AP-1A and AP-1B in retrograde transport. For a more detailed discussion of polarized sorting mediated by AP-1, we refer to an excellent review (Bonifacino, 2014).

In other cells, such as endothelial cells, AP-1 has been reported to be involved in the formation and/or maturation of Weibel-Palade bodies and immature secretory granules (Lui-Roberts et al., 2005; Nass et al., 2021).

### epsinR

Another clathrin adaptor operating at early endosomes apart from AP-1 is epsinR (derived from epsin-related) (Figure 1A; Figure 2). EpsinR, also termed CLINT1, epsin4 or enthoprotin, is a monomeric adaptor protein of ∼70 kDa with an epsin N-terminal homology (ENTH) domain, a structural domain that is present in epsins operating in CME (Bonifacino and Traub, 2003). The epsin family does not only include epsinR and the plasma membrane epsins 1–3, but also tepsin. Tepsin was described as the first accessory protein of AP-4, supporting the notion of a general function of epsins in AP-mediated protein traffic (Borner et al., 2012; Mattera et al., 2015; Archuleta et al., 2017; Mattera et al., 2020).

EpsinR was originally discovered in a pulldown screen for proteins interacting with the appendage domain of the γ-subunit of AP-1 (Hirst et al., 2003; Mills et al., 2003). Since epsinR interacts with AP-1 *in vitro* and *in vivo*, it was not surprising that they have a nearly identical intracellular distribution pattern. Association of epsinR with membranes, however, is independent of AP-1, since epsinR localizes normally in AP-1-deficient cells. Likewise, AP-1 does not depend on epsinR for proper localization (Hirst et al., 2003). Similar to AP-1, epsinR is recruited to membranes by members of the ARF GTPase family and the phosphoinositide PI(4)P (Hirst et al., 2003).

What is the function of epsinR in retrograde transport? The first cargo that was found to bind epsinR in a yeast two-hybrid screen was the SNARE vti1b (Chidambaram et al., 2004), an interaction that was subsequently corroborated *in vivo* (Hirst et al., 2004) and analyzed more molecularly in detail (Miller et al., 2007). It was shown that depletion of epsinR altered the steady-state distribution of vti1b as well as of vti1a, a SNARE which is 33% identical to vti1b, from a perinuclear to a more scattered peripheral localization pattern. Since vti1b was also strongly reduced in CCV fractions isolated from epsinR-depleted cells, epsinR was characterized as a SNARE-specific cargo adaptor that most likely operates in endosome-to-TGN retrieval of vti1b.

That epsinR/clathrin is not exclusively acting as SNARE-specific adaptor, but functioning in endosome-to-TGN cargo transport more generally was demonstrated (Saint-Pol et al., 2004). Using a sulfation assay, the study showed that cells depleted of epsinR had deficits in delivering Shiga toxin, CIMPR, as well as TGN38/46 from TfR-positive compartments to the TGN (Saint-Pol et al., 2004). EpsinR was also shown to regulate efficient transport of Shiga toxin from endosomes to the TGN (Saint-Pol et al., 2004; Selyunin and Mukhopadhyay, 2015).

Recently, an epsinR knocksideways cell line has been described to analyze the immediate consequences of epsinR inactivation on clathrin-mediated intracellular traffic (Hirst et al., 2015a). Similar to the authors’ former knocksideways study (Hirst et al., 2012a), they isolated CCVs from cells where epsinR was rapidly inactivated, followed by quantitative CCV proteome analysis. Surprisingly, it was found that the epsinR knocksideways had a more global effect on intracellular CCV cargoes, similar to the effect of an AP-1 knocksideways. Top hits of depleted proteins were not only epsinR itself and predicted cargo proteins (e.g., vti1b), but also other coat components, suggesting that, like AP-1, epsinR plays a critical role in the formation of an entire CCV population (Hirst et al., 2015a). Interestingly, several cargo proteins that depend on AP-1 and/or GGAs (e.g., hydrolase receptors) were depleted even more strongly from CCVs isolated from epsinR than from AP-1 knocksideways cells. It thus might be that epsinR and AP-1 belong to the same retrograde transport pathway, apart from their independent cellular functions. That their action must not be exclusively cooperative was evidenced by the finding that depleting epsinR or AP-1 with RNAi produced different effects on retrograde transport of overexpressed CDMPR. While epsinR knockdown decreased the transport rate of CDMPR to the TGN, AP-1 inactivation caused the opposite (Buser et al., 2022).

### GGA1–3

Adaptor protein localization to endosomes may suggest a function in cargo recycling to the plasma membrane or retrieval to the TGN. Another cargo adaptor molecule localizing to endosomes are the Golgi-localized, γ-ear-containing, ADP ribosylation factor (ARF)-binding proteins, commonly known as GGAs (Bonifacino, 2004). Localization of GGAs to endosomes might sound odd since they have been reported to mainly act from Golgi membranes to sort cargo, in particular with AP-1 (Puertollano et al., 2001a; Puertollano et al., 2001b; Doray et al., 2002). Since that time AP-1 has been primarily considered a cargo adaptor regulating anterograde transport from the TGN, hence GGAs have been naturally linked to operate from the same compartment. Evidence of AP-1 function in retrograde transport from endosomes have emerged only after the description of fibroblasts deficient of μ1A (Meyer et al., 2000), opening the question whether all observed and described AP-1/GGA-positive membrane structures since were indeed of TGN identity.

Over the last decades, several findings by independent laboratories pointed towards GGA involvement in retrograde traffic from endosomal membranes. For example, it was shown that GGA1, one out of three GGAs (GGA1-3), promotes retrograde transport of the processing enzyme BACE1 from early endosomes to the TGN (Wahle et al., 2005). Moreover, a recent study performed in *Schizosaccharomyces pombe* demonstrated that GGAs in collaboration with clathrin adaptors indeed contribute to efficient retrograde transport of Vps10, yeast’s MPR homologue, from the prevacuolar endosome to the TGN (Yanguas et al., 2019), again highlighting the evolutionary conservation of the mechanistic basis of this transport route. Some other lines of evidence confirming GGA localization and function on endosomes were reported, but not for function in retrograde transport (D'Souza et al., 2014). In that study, the authors showed that GGA3 localizes onto dynamic Rab4-enriched tubular domains of early endosomes, thereby potentially regulating recycling to the cell surface. The possibility, however, that GGAs could also operate in retrograde transport of MPRs has been only addressed in mammalian cells recently. Knocking down all three GGAs decreased the rate of transport of CDMPR to the TGN (Buser et al., 2022), in line with a role of these adaptors in retrograde traffic from endosomes. This finding was supported by the observation that silencing of GGAs produce a similar MPR dispersal phenotype as reported for AP-1 (Ghosh et al., 2003b; Buser et al., 2022). Interestingly, CRISPR/Cas9-engineered triple GGA1–3 knockout cells showed a redistribution of CIMPR from peripheral punctae to the TGN (Doray et al., 2021), contrasting previous findings (Ghosh et al., 2003b).

GGAs clearly localize both to the TGN and to endosomes (Dell'Angelica et al., 2000; Hirst et al., 2000; Ghosh et al., 2003b; Wahle et al., 2005; Ratcliffe et al., 2016; Uemura et al., 2018; Uemura and Waguri, 2020), like AP-1, and thus might operate at both places. Comparative CCV proteomics with GGA2 and AP-1 knocksideways cells pointed towards involvement of GGA/AP-1 coats in anterograde sorting of MPR–lysosomal hydrolase complexes from the TGN (Hirst et al., 2012b), yet the authors did not discount a potential retrograde function. A very surprising finding by Hirst et al. (2012b) was that Rabaptin5 (RABEP1) was the only known accessory component to be significantly lost from GGA2 knocksideways CCVs. Rabaptin5 is a marker of early endosomes where, as a complex with Rabex5 it activates Rab5 (Kalin et al., 2016). The fact that GGA depletion affects the CCV association of an endosomal protein points towards a role of these adaptors on endosomes, possibly in retrograde transport. More direct experiments focusing on retrograde transport mediated by GGA adaptors are required to solve the puzzle about their directionality at the TGN-to-endosome interface.

### AP-5-dependent pathway

As AP-1, AP-5 is a member of the heterotetrameric cargo adaptor protein (AP) complex family and localizes to late endosomes (Figure 1A; Figure 2). Unlike to AP-1 and AP-2, transport carriers nucleated by AP-5 do not rely on clathrin and ARF GTPase for their formation (Hirst et al., 2011; Hirst et al., 2012b). AP-5 is also less abundant than AP-1 and AP-2, with about 12,000 copies per cell (Hirst et al., 2012b). In contrast to the clathrin-dependent AP complexes, AP-5 is stably associated with two additional proteins, proteins SPG11 (spatacsin) and SPG15 (spastizin). Recruitment of AP-5 onto endosomal membranes is dependent on phosphatidylinositol 3-phosphate (PI3P) (Hirst et al., 2013). Recently, the machinery regulating recruitment of AP-5/SPG11/SPG15 to late endosomes/lysosomes has been described. Levels of PI3P as well as Rag GTPases are critical in regulating localization of AP-5 to membranes (Hirst et al., 2021). Reduction of AP-5 by RNAi caused swelling of multivesicular bodies (MVBs) with CIMPR-positive tubules emanating from them, indicating a potential role in recycling (Hirst et al., 2015b; Hirst et al., 2018). In general, fibroblasts deficient for the ζ-adaptin of AP-5, SPG11, or SPG15 show aberrant endolysosome morphology, suggesting that endosome/endolysosome maturation might be altered.

Antibody uptake immunolocalization assays showed that adaptor loss leads to impaired retrieval of CIMPR to a TGN46-positive compartment, suggesting a role of AP-5 in retrograde transport (Hirst et al., 2018). In search for additional AP-5 dependent cargo proteins, isolation of vesicle fractions from knockout cells revealed several Golgi-resident transmembrane proteins with diverse topologies. Recycling of these Golgi proteins is not mediated by direct binding of their short cytoplasmic tails to the adaptor, but by binding to the multipurpose cargo receptor sortilin and potentially other members. AP-5 is thus involved in endosome-to-Golgi retrieval of CIMPR and sortilin family receptors.

The study of AP-5 is of particular interest since it is linked to hereditary spastic paraplegia, a disorder where patients suffer from progressive spasticity of the lower limbs with a relatively early age of onset, but additionally many suffer mild intellectual disability with learning difficulties in childhood and/or progressive cognitive decline (Hirst et al., 2012b; Sanger et al., 2019). The mechanistic interplay of Rag GTPases with AP-5/SPG11/SPG15 and links to mTORC1 helped to explain deficiency phenotypes, including the defect in autophagic lysosome reformation (Hirst et al., 2021).

### Retromer- and SNX-dependent pathways

The retromer complex is an evolutionary conserved multimeric protein coat that is considered a master conductor in the orchestration of multiple cargo sorting events within the tubular endosomal network (TEN) (Bonifacino and Hurley, 2008; Cullen and Korswagen, 2011; Seaman, 2012; Gallon and Cullen, 2015; Mukadam and Seaman, 2015) (Figure 1A; Figure 2). Unlike the classical coats, such as COPI, COPII, or clathrin, the retromer complex does not form a visible electron dense layer on membranes by electron microscopy (Arighi et al., 2004; Seaman, 2004; Popoff et al., 2007). Even though the terminology ‘retro’-mer complex suggests its exclusive role in retrograde transport, it actually facilitates also endosomal cargo recycling to the plasma membrane (Gallon and Cullen, 2015; Wang et al., 2018).

Though conserved between kingdoms, the retromer complex in mammalian cells features some subtle functional and structural particularities that yeast do not have. The retromer complex was initially identified more than two decades ago in *Saccharomyces cerevisiae* to be required for endosome-to-TGN retrieval of the carboxypeptidase Y (CPY) receptor Vps10, ‘yeast’s MPR’. In yeast, the retromer complex is made up of two different subcomplexes, a heterotrimer of Vps26, Vps29, Vps35, and a heterodimer of Vps5 and Vps17 (Horazdovsky et al., 1997; Seaman et al., 1997; Seaman et al., 1998). In mammalian cells, genes encoding Vps5 and Vps17 have diversified such that the sorting nexin 1 and sorting nexin 2 (SNX1 and SNX2) are the mammalian homologues of Vps5, while SNX5 and SNX6 are counterparts of Vps17. Any combination of SNX1 or SNX2 with SNX5 or SNX6 can assemble to the heterodimeric subcomplex (Rojas et al., 2007; Wassmer et al., 2007).

A particular feature of these SNXs is that they comprise a C-terminal Bin/Amphiphysin/Rvs (BAR) domain, and hence are termed SNX-BARs (Carlton et al., 2004). These BAR domains enable SNX-BARs to form specific homo- and heterodimers and, in this structural arrangement, can sense, model and drive membrane curvature for the formation of tubular-vesicular carriers (Zimmerberg and McLaughlin, 2004; Frost et al., 2008; Frost et al., 2009; van Weering et al., 2012). In addition, SNX-BARs of the retromer subcomplex also comprise a phagocytic oxidase (phox) homology (PX) domain (Teasdale and Collins, 2012), a domain specifically binding to PI(3)P-enriched membranes. As a result of coincidence detection of PI(3)P and curvature, SNX-BARs preferentially associate with tubular-vacuolar membranes of early endosomes. The heterodimeric subcomplex in its organization and function is often referred to as the SNX-BAR subcomplex or the ‘tubulation complex’ (Gallon and Cullen, 2015; Trousdale and Kim, 2015).

Considering the retromer complex as two distinct subcomplexes is often necessary. Then even though the SNX-BAR subcomplex interacts strongly with the heterotrimeric subcomplex in yeast, this interaction appears to be less robust in mammalian cells. It thus seems that the two subcomplexes in mammals only transiently interact with each other, similar to some AP complexes with clathrin on the respective membranes during carrier formation. Since the heterotrimeric Vps26-Vps29-Vps35 subcomplex together with various other factors select cargo for transport, it is often referred to as ‘cargo selective complex (CSC)’, ‘cargo recognition complex (CRC)’, or ‘retromer’. We will use the terminology retromer and, hence, retromer and the SNX-BARs form together the ‘retromer complex’.

Retromer cannot bind to PI(3)P-enriched early endosomes on its own since it lacks a lipid-binding domain. Instead, retromer requires Rab7a for membrane recruitment, most probably *via* Vps35 (Nakada-Tsukui et al., 2005; Rojas et al., 2008; Seaman et al., 2009). As commented elsewhere (Johannes and Wunder, 2011), this finding is rather puzzling since Rab7 is associated with late rather than early endosomes. It is thus believed that cargo sorting by retromer complex is a progressive process that is part of endosomal maturation during the Rab5-to-Rab7 switch (Rojas et al., 2008). Along with Rab7a, SNX3 has also been implicated in the recruitment of retromer (Harterink et al., 2011). Unlike the SNX-BARs, SNX3 belongs to the SNX-PX subfamily of SNXs since it only has a PX, but not a BAR domain (Yu and Lemmon, 2001; Gallon and Cullen, 2015). Unlike the ‘canonical retromer complex’, the SNX3-retromer complex represents a heterotetrameric and not a heteropentameric assembly complex. SNX3 has been reported in the selective transport of WLS or APP to the TGN (Harterink et al., 2011; Vardarajan et al., 2012).

Compared to retrograde transport mediated by AP-1 and other clathrin adaptors, retromer complex-driven sorting is probably the most thoroughly characterized retrograde endosome-to-TGN pathway. Therefore, it is not surprising that a number of cargo proteins have been described that are sorted by the retromer complex. Probably the best-characterized cargo of the SNX-BAR retromer complex is CIMPR. In a previous study (Seaman, 2004), it was questioned whether the mammalian retromer complex fulfills the same function as in yeast regarding endosome-to-TGN retrieval of CIMPR. Using cells derived from transgenic mice deleted for mammalian Vps26 and through the application of RNAi to knockdown Vps26, it was found that retromer subunit depletion resulted in a range of phenotypes consistent with a defect in endosome-to-Golgi retrieval. Similar to a knockdown or knockout of AP-1 (Meyer et al., 2000; Hirst et al., 2005), Vps26 depletion caused CIMPR redistribution to EEA1-positive endosomes and defects in cathepsin D maturation (Seaman, 2004). A parallel and independent study reported similar phenotypes for cells depleted of Vps35 (Arighi et al., 2004). It thus seems that ligand-free CIMPR, or MPRs in general, is dependent on more than just one retrograde sorting machinery. Apart from MPRs, the other two listed cargo receptors, sortilin and SorLA, are also trafficked from endosomes to the TGN in a SNX-BAR-retromer-dependent manner (Seaman, 2004; Nielsen et al., 2007; Fjorback et al., 2012).

Other endosome-to-TGN cargoes sorted by retromer complex are DMT1-II, TGN38 and WLS (Belenkaya et al., 2008; Tabuchi et al., 2010; Bai and Grant, 2015). However, unlike MPR and MPR-like cargo receptors, DMT1-II, TGN38 and WLS transport is mediated by the SNX3-retromer complex. Also here, it has to be mentioned that TGN38 is also recycled to the TGN by an alternative route, namely, by the epsinR pathway (Saint-Pol et al., 2004).

As briefly touched above, some cargo proteins (e.g., MPRs or TGN38 and isoforms) seem to use more than just one transport route for correct membrane localization. Together with the fact that retromer complex has been appreciated not only as cargo coat but also as ‘recruiting hub’ for multiple factors, the idea has raised that retromer complex is linked to clathrin coat formation on endosomes (Seaman, 2012; Burd and Cullen, 2014; Gallon and Cullen, 2015). Clathrin-coated structures on endosomes in close vicinity to retromer complex have been indeed reported, and proteomics-based studies have identified retromer complex subunits to be present in crude preparations of CCVs (Borner et al., 2006; Popoff et al., 2007; Popoff et al., 2009; Shi et al., 2009). In contrast to these observations, however, it was also reported that clathrin and retromer complex subunits are present on distinct transport intermediates (Borner et al., 2012). In addition to that, there are currently no reports of any direct interactions between clathrin and retromer or the SNX subunits (McGough and Cullen, 2011; McGough and Cullen, 2013). Thus, whether clathrin and retromer complex operate together in endosomal protein sorting requires further investigation.

Similar to other cargo coats such as AP-1, retromer complex selectively recognizes short linear amino acid stretches in the cytoplasmic tail of transmembrane proteins. Vps35 is thought to select cargo, specifically CIMPR, by association with a WLM motif (Seaman, 2007) (Figure 1B). Similarly, Vps26 binds the sequence FANSHY in the cytoplasmic tail of SorLA (Fjorback et al., 2012). Several lines of evidence have indicated that SNX components might also be involved in cargo recognition (Strochlic et al., 2007; Harterink et al., 2011; Temkin et al., 2011; Zhang et al., 2011; Steinberg et al., 2013). It was assumed that the recognition might depend either on posttranslational or structural motifs as no generic amino acid motifs acting as cargo sorting determinants could be determined. Recently, however, the concept of retromer acting as cargo recognition device has been critically challenged. Not the Vps26-Vps29-Vps35 subcomplex, but the SNX-BAR dimers associate with the WLM motif for endosome-to-Golgi retrieval (Figure 1B) (Kvainickas et al., 2017; Simonetti et al., 2017; Simonetti et al., 2019; Yong et al., 2020). Furthermore, these studies suggested that retromer was not required for CIMPR retrieval, thereby contradicting the established literature. To exclude any indirect effects by gradual or long-term depletion, a system to acutely inactivate retromer was established (Evans et al., 2020). Rapid rerouting of retromer *via* Vps35 to non-secretory compartments led to time-resolved GLUT1 sorting defects, but not to any alterations in CIMPR retrograde trafficking. Due to the emerged and predominant role of SNX-BARs, particularly of SNX1/SNX2-SNX5/SNX6, in sorting, this sorting complex is referred to as ‘Endosomal SNX-BAR sorting complex for promoting exit-1’ (ESCPE-1). Other combinations of SNXs lead to additional ESCPE complexes.

There are some similarities between the sorting motifs recognized by the different SNX proteins. Both SNX5 and SNX6 can sort the CIMPR *via* the WLM motif and SNX3 can bind to the DMT1-II tail *via* the YLL motif (Figure 1B). Currently, it is not known which machinery sort sortilin *via* its FLV motif. Since the WLM and YLL motifs are biochemically very similar to the FLV motif, SNX3 or SNX5/SNX6 could be possible candidates. Thus, it would follow that SNX3 could also sort CIMPR *via* the WLM motif in conjunction with Vps26-Vps29-Vps35, and that SNX5 and SNX6 could sort DMT1-II (Seaman, 2007; Tabuchi et al., 2010; Lucas et al., 2016; Cui et al., 2019).

The finding of different readouts by inactivating the same machinery was surprising as different laboratories have reproduced impaired CIMPR retrieval phenotypes by retromer inactivation (Arighi et al., 2004; Seaman, 2004; Wassmer et al., 2007; Harbour et al., 2010; Hao et al., 2013; McGough et al., 2014; Follett et al., 2016; Cui et al., 2019), while others could not (Kvainickas et al., 2017; Simonetti et al., 2017; Simonetti et al., 2019). Prompted by the description of SNX-BARs operating as device in endosome-to-Golgi retrieval, many comments, reports and reviews have been published summarizing the challenged view and controversies on retromer complex and SNX-BARs (Chen et al., 2019; Seaman, 2021; Tu and Seaman, 2021). We refer the reader to these resources for a more detailed discussion of this controversy. Most, if not all, of the studies cited above applied immunofluorescence microscopy combined with colocalizaton anaylsis to report their findings. In other studies (Buser et al., 2018; Buser et al., 2022), a biochemical approach based on cargo surface labeling with sulfation-competent nanobodies was applied to monitor TGN arrival. This method allows the ease quantification of arrived receptor in the TGN by autoradiography or scintillation. Using this approach for CDMPR, a clear reduction of receptor arrival in the TGN was observed in Vps26a-depleted cells (Buser et al., 2022), suggesting retrieval function of retromer for CDMPR. This finding contrasts a previous report showing CDMPR to be a retromer-independent cargo (Cui et al., 2019). Sorting determinants in the cytoplasmic tail of CDMPR for retromer remain unknown. Further, applying the nanobody approach for CIMPR in Vps26- or ESCPE component-depleted cells would be interesting to disentangle controversies in the field of cargo recognition by retromer and SNX-BARs.

### Rab9/TIP47-dependent pathway

The first discovered pathway that mediates retrograde transport from an endocytic compartment to the TGN in mammalian cells was not through AP-1 or the retromer complex, but the Rab9/TIP47 pathway (Figure 1A; Figure 2) (Pfeffer, 2009). The GTPase Rab9 has been shown to localize to tubular late endosomes and to be required for efficient transport of MPRs to the TGN (Lombardi et al., 1993; Soldati et al., 1993). With the subsequent search for additional factors binding Rab9, a protein of 47 kDa, named tail-interacting protein, briefly TIP47, was found by a yeast two-hybrid screen (Diaz and Pfeffer, 1998). In a follow-up study, it was then shown that Rab9 and TIP47 are operating in an intertwined process where active Rab9 acts as crucial hub to recruit downstream effectors, including TIP47, to mediate late endosome-to-TGN transport (Carroll et al., 2001).

Additional characterization demonstrated that depletion of TIP47 using antisense oligonucleotides or siRNA strongly destabilized MPRs in living cells (Diaz and Pfeffer, 1998; Ganley et al., 2004), and antibody depletion of TIP47 from cytosol led to a partial loss of cytosol activity in terms of its ability to support *in vitro* transport of MPRs from endosomes to the TGN (Diaz et al., 1997). The association of TIP47 with purified endosome-enriched membranes was impaired by antibodies binding to the cytoplasmic domain of MPRs, implicating TIP47 to specifically recognize MPRs (Krise et al., 2000; Orsel et al., 2000). Also, binding of TIP47 to CIMPR was reported to be somewhat stronger than to CDMPR, suggesting small trafficking differences between the two receptors in retrieval (Krise et al., 2000) (Figure 1B). TIP47 expression was also shown to stimulate MPR transport from late endosomes to the TGN, and the presence of Rab9 even increased the affinity with which TIP47 bound MPR tails (Sincock et al., 2003). All these findings led to the proposal of a model where Rab9-recruited TIP47 acts as a cargo selection device for MPRs in late endosome-to-TGN retrieval (Pfeffer, 2009). Apart from TIP47, other Rab9 effectors are required for efficient MPR retrieval from late endosomes to the TGN, including p40 (RABEPK) and GCC185 (Diaz et al., 1997; Derby et al., 2007). While p40’s function in transport is unclear, GCC185 has been shown to be a tether for vesicle docking and fusion at the TGN.

While clathrin adaptors and the retromer complex sort a broad spectrum of cargo for retrograde transport from endosomes, TIP47 seems to specifically traffic only MPRs. Despite the considerable body of evidence, the role and function of TIP47 in cargo traffic from late endosomes has been challenged (Bulankina et al., 2009). In particular because TIP47 has also been reported in lipid droplet biosynthesis (Wolins et al., 2001), which is considered its major cellular role to date. A direct function of TIP47 in retrograde traffic of MPRs to the TGN is still under debate. Compared to Vps26 depletion where CIMPR turnover is rather fast (50% turnover in ∼3 h), antisense-mediated TIP47 depletion only produced a slow receptor turnover (50% turnover in ∼14 h) (Diaz and Pfeffer, 1998; Arighi et al., 2004; Seaman, 2004). This finding among contrasts TIP47 to act as *bona fide* machinery conferring traffic to the TGN. The role of Rab9 in MPR retrieval, however, should not be discounted. In a recent study, the function of Rab9 in retrograde traffic has been readdressed (Buser et al., 2022). Knocking down Rab9a significantly impaired endosome-to-TGN transport of CDMPR, suggesting Rab9 to be involved in this pathway.

The full repertoire of molecular factors involved in Rab9-dependent retrograde traffic remains elusive. Interestingly, despite the existence of two isoforms, Rab9a and Rab9b, most studies have focused on Rab9a. Additional studies involving Rab9 are required to understand its role in MPR recycling to the TGN. Constitutively active and dominant-negative Rab9 mutants can help to further dissect its role in the endosomal pathway (Kucera et al., 2016a; Kucera et al., 2016b). Interestingly, other Rabs, including, Rab7b and Rab29, have been reported to affect endosome-to-Golgi retrieval of receptors (Progida et al., 2010; Progida et al., 2012; Wang et al., 2014). Consistent with these findings, Rab9 is not evolutionary conserved in all metazoans.

To which extent all the discussed sorting machineries operate together or in parallel remains elusive. Knocking down or out one machinery might upregulate cargo loading by another to compensate. Using acute depletion techniques allowing specific inactivation of one or more machineries could address these shortcomings.

## Approaches to study plasma membrane-to-TGN transport

Several approaches have been established to dissect endosome-to-Golgi retrieval. The most prominent ones are based on antibody uptake followed by immunofluorescence staining. Biochemical assays using sulfation, particularly tyrosine sulfation, have been applied frequently as well. Sulfation, using the radioactively-marked sulfur nuclide ^35^S, is extremely powerful since it allows a more direct way to assay TGN arrival than microscopy-based techniques can do: It measures specifically TGN lumen arrival of proteins since sulfation is a posttranslational modification (PTM) restricted to this compartment. Tyrosine sulfation, conferred by tyrosylprotein sulfotransferases 1 and 2 (TPST1 and TPST2) and transporters for 3'-phosphoadenosine-5'-phosphosulfate (PAPST1/SLC35B2 and PAPST2/SLC35B3) (Huttner, 1988), is robust and barely influenced by inactivation of retrograde transport machinery (Buser et al., 2022). In the following, we will outline some of the techniques which have been applied to analyze endosome-to-TGN traffic.

### Tyrosine sulfation

Sulfation is not the latest developed approach to assay cell surface/endosome-to-TGN traffic. Already decades ago, several groups have independently designed tools based on sulfation site sequences that can be either expressed as tag part of a recombinant cargo or chemically coupled to protein (Sandvig and van Deurs, 1994; Johannes et al., 1997; Rapak et al., 1997; Mallard et al., 1998; Amessou et al., 2006). Particularly, recombinant fragments of ricin and Shiga toxin subunits were modified with tyrosine sulfation (TS) consensus sequences (Johannes et al., 1997; Rapak et al., 1997; Mallard and Johannes, 2003; Utskarpen et al., 2006; Shiba et al., 2010). Using these recombinant toxin subunits, it could be shown that these proteins were sulfated, revealing their passage through the TGN en route to the ER. Transplantation of TS consensus motifs was not only beneficially applied to study TGN arrival, but also to analyze TGN exit by labeling recombinant proteins from the biosynthetic pathway (Leitinger et al., 1994; Leitinger et al., 1995).

Instead of TS-tag incorporation in the protein’s amino acid sequence, others pioneered sulfation peptide chemistry approaches to cell surface-label proteins or antibodies (Saint-Pol et al., 2004; Amessou et al., 2006; Amessou et al., 2007; Christiano et al., 2010). Coupling of TS-containing peptides to anti-GFP or anti-TGN46 antibodies allowed to study retrograde transport of recombinant GFP-CIMPR or endogenous TGN46 (Saint-Pol et al., 2004). In this study, this approach demonstrated epsinR to be partly involved in endosome-to-TGN transport of CIMPR and TGN46. Using such an antibody-based approach to monitor retrograde traffic brings along many technical and experimental caveats, such as antibody-mediated receptor crosslinking.

Another sulfation-based approach to study retrograde transport of receptor proteins from the cell surface to the TGN has been used (Sincock et al., 2003; van Rahden et al., 2012). Instead of using a cell surface label (e.g., TS-tagged antibodies), the protein of interest is directly tagged with a site conferring tyrosine sulfation. To detect endosome-to-TGN traffic, cells of interest expressing TS-tagged reporters are first incubated in sulfate-free medium containing excess of chlorate to prevent sulfation of newly synthesized proteins. Chlorate is a reversible inhibitor of sulfation (Humphries and Silbert, 1988; Safaiyan et al., 1999) and competitively interferes with the formation of 3'-phosphoadenosine-5'-phosphosulfate (PAPS). The reporter is then chased to its steady-state localization, while new synthesis is blocked with the addition of cycloheximide. After chlorate removal, reporter transport from endosomes to the TGN is then measured by incubating cells in the presence of radiolabeled sulfate and cycloheximide. An approach in this setup has been used to monitor retrograde transport defects of CDMPR when endosomal OCRL phosphatase (van Rahden et al., 2012) and TIP47 (Sincock et al., 2003) have been depleted. The disadvantage of this experimental strategy is that one has to chemically block sulfation by adding excess of chlorate in the presence of cycloheximide.

To bypass some of the shortcomings of these sulfation-based approaches, especially TS site-modified antibodies, the use of functionalized and bacterially expressed anti-GFP nanobodies proved beneficial (Buser et al., 2018). Derivatizing such monovalent protein binders with a TS site allowed their specific uptake by GFP-modified proteins at the cell surface and their piggyback transport to the TGN. Sulfated nanobodies can be recovered from lysed cells using purification resins, followed by analysis using autoradiography. Using this approach, endosome-to-TGN traffic of CIMPR and CDMPR could be assessed in machinery-depleted cells (Buser et al., 2018).

An alternative, radiolabel-free approach to study TGN arrival is by using anti-sulfotyrosine antibodies in combination with the nanobody approach (Buser et al., 2018). The only existing and commercial antibody detecting sulfotyrosine as epitope independent of sequence context has been described (Hoffhines et al., 2006), yet the sensitivity is reduced compared to radiolabeling.

### Resialyation

An other approach to assess PM-to-TGN transport has been initially described is oligosaccharide resialyation (Duncan and Kornfeld, 1988). The assay relies on oligosaccharide processing of retrogradely transported cargo proteins. To do so, mature oligosaccharides units of cell surface receptors are enzymatically desialylated so that they serve as substrates for trans-Golgi/TGN-sialyltransferases. If the deglycosylated receptor then returns to this compartment, the oligosaccharides regain radiolabeled sialic acid. This approach and variants thereof have been widely used in the past (Goda and Pfeffer, 1988; Goda and Pfeffer, 1989; Jin et al., 1989; Draper et al., 1990; Prydz et al., 1990; Riederer et al., 1994). This kind of assay was applied to examine the involvement of AP-1 in MPR retrieval to the TGN (Meyer et al., 2000).

### Proteomics

Proteomics has gained fundamental importance over the last decades, and therefore it is not surprising that attempts have been undertaken to analyze retrograde transport using the power of mass spectrometry. Previously, a SNAP-tag-based proteomics approach to study cell surface-to-TGN transport of endogenous proteins was presented (Shi et al., 2012). The authors created a TGN-localized trap composed of truncated GalT fused to GFP and a SNAP-tag. Cargo that has been chemically cell surface-labeled with benzylguanine (BG) can react and then be covalently linked to the recombinant trap if retrograde transport to the TGN has occurred. Applying this approach, the authors (Shi et al., 2012) could present a list of 20 proteins, including GPCRs, transporters, kinases and more, that undergo retrograde traffic to the TGN. Among the hits, TfR, the first proposed endogenous retrograde cargo protein (Snider and Rogers, 1985), was detected, too. This observation is in contrast to the general notion that recycling receptors, such as TfR or ASGPR, reach the Golgi. It has been reported, though, that glycosyltransferases can exit the TGN to some extent as well (Hathaway et al., 2003; Podinovskaia et al., 2021; Sun et al., 2021). This can potentially explain why TfR was found crosslinked to the trap. Surprisingly and interestingly, the SNAP-tag-based proteomics approach failed to detect MPRs and TGN46 (Shi et al., 2012). These results imply that the mass spectrometry methodology can also pass over relevant candidates that are amenable with classical biochemical approaches. A similar SNAP-tag-based approach to investigate retrograde transport to the ER has been described (Geiger et al., 2012). Nevertheless, it might be about time to revisit this approach.

In recent years, mass-spectrometry has become more sensitive, and new methodologies to enrich for specific intracellular compartments are available. Recently, another proteomics approach based on rerouting and capturing of endosome-derived vesicles on mitochondria *via* golgin tethers has been reported (Shin et al., 2020). Using this approach, cargo in endosome-derived vesicles captured by specific golgins could be identified.

### Microscopy

The ease of image analysis of stained samples have made microscopy an ideal tool to study endosome-to-Golgi retrieval of proteins. Compared to other techniques described above, microscopy has the advantage of being in general more rapid, straightforward, and cheaper. Labeling of the surface pool of an endogenous or recombinant cargo with an antibody that detects the lumenal portion of the protein, followed by uptake and colocalization analysis of antibody with a TGN marker protein, represents a prominent assay to assess Golgi arrival. Antibody uptake experiments of this kind have been applied to study impairment of retrieval in the absence of AP-1, Vps26, or Rab9 (Meyer et al., 2000; Robinson et al., 2010; Chia et al., 2011). Recently, such an image-based antibody uptake approach has been applied to screen for factors required for endosome-to-Golgi retrieval of CIMPR (Breusegem and Seaman, 2014). The authors used the chimeric reporter CD8-CIMPR whose surface pool could be labeled with anti-CD8 antibodies and traced back to the Golgi which has been labeled with GFP-GOLPH3. Using this approach, not only established factors could be confirmed, but also a number of multipass membrane-spanning proteins were shown to be required for efficient endosome-to-TGN delivery of CIMPR (Breusegem and Seaman, 2014).

Instead of using antibody uptake experiments, often receptor protein dispersal phenotypes have been employed to assess retrograde transport defects mediated by machinery. In particular for the MPRs, redistribution of receptor molecules from juxtanuclear to more peripheral compartments can be readily assessed and quantified. MPR dispersal phenotypes are often quantified by monitoring the colocalization of tagged or endogenous MPR with endogenous endosomal markers, such as EEA1 for instance. Dispersal phenotype analysis have been used as experimental argument in the retromer/SNX controversy (Arighi et al., 2004; Seaman, 2004; Wassmer et al., 2007; Kvainickas et al., 2017; Simonetti et al., 2017; Tu and Seaman, 2021). Other fluorescence-based approaches, such as flow cytometry, have been shown useful to assess intracellular transport of proteins (Chia et al., 2014).

## Conclusion and perspectives

Over the last decades, a considerable progress has been made in identifying machineries and factors involved in endosome-to-Golgi retrieval of cargo proteins. Responsible for this progress are on the one hand more advanced and sophisticated technologies, in particular in the field of microscopy, and on the other hand the use of high-throughput siRNA and CRISPR/Cas9 applications to globally screens for factors pertubating traffic at the endosome-to-TGN interface. Giving renaissance to ‘old-fashioned’ techniques such as radiolabeling of proteins using sulfation can provide additional information to reconcile image-based approaches. Unfortunately, most of our knowledge of retrograde transport machineries has been based on a few model proteins, while the machinery for a plethora of cargo proteins must be still characterized. Additionally, some of these retrograde transport machineries seem to work redundantly, indicating some physiological relevance. On the bright side: there is still a lot of interesting biology awaiting discovery!
